# An Involvement of Oxidative Stress in Endoplasmic Reticulum Stress and Its Associated Diseases

**DOI:** 10.3390/ijms14010434

**Published:** 2012-12-24

**Authors:** Bidur Bhandary, Anu Marahatta, Hyung-Ryong Kim, Han-Jung Chae

**Affiliations:** 1 Department of Pharmacology, School of Medicine, Chonbuk National Univeristy, Jeonju 561-180, South Korea; E-Mails: bidurbhandary@gmail.com (B.B.); marahattaanu@gmail.com (A.M.); 2 Department of Dental Pharmacology, Dental School, Wonkwang University, Iksan 570-749, South Korea

**Keywords:** ER stress, ER stress associated disease, ER associated oxidative stress, disulfide bond formation, PDI, ERO-1α, mitochondria electron transport chain

## Abstract

The endoplasmic reticulum (ER) is the major site of calcium storage and protein folding. It has a unique oxidizing-folding environment due to the predominant disulfide bond formation during the process of protein folding. Alterations in the oxidative environment of the ER and also intra-ER Ca^2+^ cause the production of ER stress-induced reactive oxygen species (ROS). Protein disulfide isomerases, endoplasmic reticulum oxidoreductin-1, reduced glutathione and mitochondrial electron transport chain proteins also play crucial roles in ER stress-induced production of ROS. In this article, we discuss ER stress-associated ROS and related diseases, and the current understanding of the signaling transduction involved in ER stress.

## 1. Introduction

**Figure 1:**
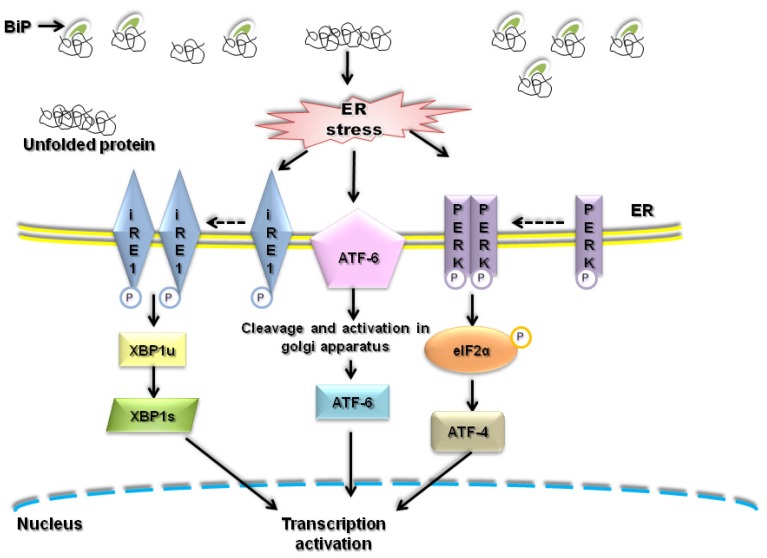
Endoplasmatic reticulum (ER) stress and unfolded protein response (UPR) induction. During the pathological conditions, proteins are aggregated causing accumulation of misfolded proteins in the ER lumen. This accumulation of misfolded proteins enhances UPR. During the initiation of UPR, BiP preferentially binds to the unfolded proteins, driving its equilibrium binding away from inositol requiring enzyme 1 (IRE-1), PKR-like endoplasmic reticulum kinase (PERK) and activating transcription factor 6 (ATF-6) proteins which are considered as initiators of the three main signaling cascades of UPR. IRE-1 protein dimerizes and activates its protein kinase activity (for autophosphorylation) and its endoribonuclease activity. It cleaves X-box-binding protein 1 (XBP1) mRNA to remove a small intron, converting unspliced (XBP1u) to spliced form (XBP1s), resulting in yielding a more potent transcriptional activator. Similarly, PERK also dimerizes and activates to phosphorylate eukaryotic initiation factor 2 (eIF2) on the α-subunit. This action selectively translates ATF-4 mRNA which further induces more transcriptional activator. Activation of ATF-6 allows it to translocate to the golgi apparatus, where it is cleaved by a protease, changing it to the active cytosolic ATF-6 fragment. This fragment migrates to the nucleus, activating the transcription of UPR target genes.

The endoplasmic reticulum (ER) is an organelle that contains protein chaperones and enzymes, which are involved in protein folding. Protein folding is a delicate process such that only properly folded proteins are modified at the Golgi apparatus and translocated to their destined sites [1]. On the other hand, misfolded or immature proteins aggregate in the ER lumen and are degraded by the ER-associated degradation (ERAD) machinery or by autophagic degradation [2]. Misfolded proteins can expose hydrophobic amino-acid domains that enhance protein aggregation [3]. These protein aggregates trigger a condition called ER stress, which is known to contribute to the pathophysiology of “conformational diseases” such as neurodegenerative disorders and diabetes mellitus. In response to ER stress, cells activate the “ER stress response” or the “unfolded protein response” (UPR) [1,4] in order to nullify the induction of stress in the ER lumen (Figure 1). Generally, the ER stress response in mammals consists of four mechanisms: (i) Attenuation of protein synthesis to prevent any further protein aggregation/accumulation, (ii) transcriptional induction of ER chaperone genes to enhance folding capacity, (iii) transcriptional induction of ERAD genes to increase ERAD ability/capacity, and (iv) induction of apoptosis to remove stressed cells [5].

Accumulating evidence has suggested a direct link between the production of reactive oxygen species (ROS) and cellular events such as protein oxidation and protein folding [6]. Oxidative stress and ROS generation are integral components of ER stress and are not just consequences of ER stress induction. The major enzymatic components of ROS production during UPR induction are protein disulfide isomerase (PDI), endoplasmic reticulum oxidoreductin (ERO-1), and NADPH oxidase complexes (especially the Nox4). Additionally, mitochondrial electron transport enzymes produce ROS [7]. Although there is a plethora of studies on ER stress and associated diseases, the core pathophysiological mechanism of ER stress-associated ROS has not yet been fully clarified. In this review, we discuss ER stress-associated ROS and related disorders, and the current understanding of the signal transduction mechanism involved in ER stress.

## 2. ER stress and ROS

### 2.1. Understanding of ROS

Cells have basal level of ROS for signaling and normal functioning. In contrast, ROS levels increase upon exposure to toxic agents such as irradiation and environmental pollutants or during enzymatic reactions (e.g., mitochondrial respiratory chain reactions, arachidonic acid pathway, cytochrome P450 family and those involving glucose oxidase, amino acid oxidase, xanthine oxidase, NADP/NADPH oxidase or NO synthases) [8,9]. The mitochondrial inner membrane potential defines the rate of electron transport chain, which produces the membrane-impermeable superoxide anion. The generated superoxide is converted to hydrogen peroxide (H_2_O_2_) by mitochondrial dismutase and diffuses out of the mitochondria into the cytoplasm. H_2_O_2_ forms the highly reactive hydroxyl radical (OH^•^) through the Fenton reaction in the presence of iron [10]. Moreover, the superoxide anion radical (O_2_^−•^) generates other toxic metabolites such as peroxynitrite (ONOO^−^), hypochlorous acid (HOCl), and singlet oxygen (O_2_) [11]. Under basal physiological conditions, endogenous antioxidant defense mechanisms including enzymatic (e.g., superoxide dismutase, glutathione peroxidase, catalase, and thioredoxin reductase) as well as non-enzymatic (e.g., vitamins) antioxidant systems prevent ROS accumulation [10,11]. Furthermore, redox homeostasis is regulated by several redox systems such as NAD^+^/NADH, NADP^+^/NADPH, and oxidized glutathione/reduced glutathione (GSSG/GSH) [8].

### 2.2. Oxidative Protein Folding in ER

Proper protein folding and formation of disulfide bonds occur in the ER. The redox status within the lumen of the ER affects protein folding and disulfide formation. The lumen of the ER, in contrast to the cytosol, has a highly oxidizing environment, with a high ratio of GSSG/GSH. Glutathione is a tripeptide (l-g-glutamyl-l-cysteinyl-glycine) that is synthesized in the cytosol. It exists in a reduced state (GSH) due to the cytosolic NADPH-dependent reaction catalyzed by glutathione reductase [12]. Interaction of GSH and thiols of proteins with ROS determines cellular redox homeostasis and its maintenance [13]. GSH acts as a major thiol-disulfide redox buffer and the GSH/GSSG ratio is used as a cellular redox state index. The ratio of reduced glutathione to oxidized glutathione is >50:1 in the cytoplasm, and 1:1 to 3:1 in the ER lumen [14]. It is well known that the oxidizing environment in the ER lumen facilitates disulfide bond formation. Additionally, the greater oxidizing environment of the ER prevents the aggregation or accumulation of unfolded proteins in the ER lumen due to its preferred oxidation state and abundant ER-resident proteins such as protein disulfide isomerase [15].

**Figure 2:**
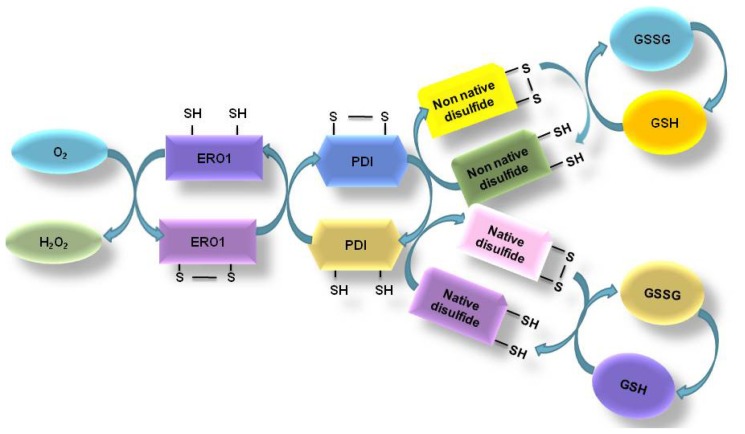
Pathway of oxidative protein folding. The formation of disulfide bonds in proteins in the ER is driven by protein disulfide isomerase (PDI) and endoplasmic reticulum oxidoreductin-1 (ERO-1). ERO-1 uses a (FAD)-dependent reaction to transfer electrons from PDI to molecular oxygen (O_2_), resulting in ER protein folding-induced oxidative stress. When incorrect disulfide bonds are formed, GSH assists in reducing them, and this decreases the GSH/GSSG ratio. This condition alters the redox environment in the ER.

Prior to the secretion of properly folded proteins from the ER lumen, these proteins undergo compulsory disulfide bond formation for stability and maturation. Alterations in the disulfide bond formation or mispairing of cysteine residues results in protein misfolding or inability of the protein to attain its proper configuration [16]. Several reports have described the role of GSH in preventing non-native disulfide bond formation or the generation of misfolded proteins. However, there are many other pathways that cells can use to maintain native protein structure. For instance, a number of folding catalysts maintained in the ER lumen, which regulate redox conditions may also facilitate the formation and isomerization of disulfide bonds [17]. In many instances, oxidative protein folding is catalyzed by a family of ER oxidoreductases including PDI, endoplasmic reticulum protein p72 (ERp72), ERp61, ERp57, ERp44, ERp29, and PDI-P5 [8]. These folding enzymes oxidize cysteine residues of nascent proteins and help proteins form correct disulfide bonds. Reduced folding enzymes are reoxidized by ERO-1, the enzyme which can use molecular oxygen as a terminal electron acceptor (Figure 2) [18].

Many studies have indicated a crosstalk between the generation of ROS and the ER stress response. Although redox imbalance can be caused by alteration in the functions of PKR-like endoplasmic reticulum kinase (PERK) and activating transcription factor 4 (ATF-4), these proteins are also responsible for the elimination of ROS through transcriptional regulation. ERO-1 usually facilitates ROS formation inside the ER [19,20]. Indeed, disulfide-bond formation involving ERO-1 contributes significantly to the total production of ROS in the cell [20]. In addition, overexpression of ERO-1 protein shifts the redox state of PDI towards the oxidized form, which also influences oxidation of PDI substrates. During disulfide bond formation, electrons pass several thiol-disulfide exchange reactions, the thiols of the substrates, PDI and ERO-1 before reaching the molecular oxygen. Incomplete reduction of oxygen results in the formation of superoxide anion radicals, which can be transformed to H_2_O_2_ or converted to other ROS. As mentioned above, the oxidative folding process is a major folding mechanism in ER physiology. ER stress-associated oxido/reduction environment may also be correlated with ER stress-associated ROS.

### 2.3. ER Associated ROS Production under ER Stress

There is disagreement on whether unfolded proteins in the ER lumen are sufficient to activate oxidative stress. Two mechanisms have been proposed for ROS generation during disulfide bond formation. First, ROS are formed as a byproduct during the transfer of electrons from protein thiol to molecular oxygen by ERO-1 and PDI. Alternatively, ROS is generated during protein misfolding due to the depletion of GSH [7,21]. After utilizing GSH, thiols are repaired enabling them to interact with ERO-1/PDI and to be re-oxidized. These steps would produce repetitive cycles of disulfide bond breakage and formation, with each cycle generating more ROS as a byproduct [6]. Thus proteins with multiple disulfide bonds may be more susceptible to generating higher levels of oxidative stress. The second mechanism argues that ROS are generated by unfolded proteins independent of the formation of disulfide bonds. Accordingly, accumulation of unfolded proteins in the ER elicits Ca^2+^ leakage into the cytosol, increasing ROS production in the mitochondria [8]. Since protein folding and refolding in the ER lumen are highly energy-dependent processes, ATP depletion consequential to protein misfolding may stimulate mitochondrial oxidative phosphorylation to increase ATP and ROS production.

ER protein oxidation and mitochondrial oxidative phosphorylation are well-described ROS sources during ER stress. Meanwhile, the role of ER stress-associated NADPH oxidase(s) as a potential ROS source is not yet fully clarified. It is of note, however, that NADPH oxidase 4 (Nox4), one of the NADPH oxidase isoforms, has recently been implicated as a possible ROS source during strokes [22]. Nox4 is also induced in human vascular smooth cells during the ER stress caused by the oxygenated lipid product, 7-ketocholesterol or tunicamycin [23]. Additionally, in endothelial cells transfected with ER-targeted fluorescent ROS sensors, Nox4 accounts for ER-associated oxidant generation in Tat protein transfection or tunicamycin-induced ER stress, but not that caused by thapsigargin or dithiothreitol [24]. Furthermore, Nox4-associated ROS alter UPR signaling and promote Ras activation (eventually activating RohA) at the cytosolic face of ER. The main functional implication of signaling is autophagy such that when either Nox4 or autophagy protein 5 (Atg5) is disabled, cells undergo apoptosis [24]. Notably, there are other studies, which have described the role of Nox4 in ROS production in various ER stress inducing disease conditions [7,25,26].

### 2.4. Mitochondria-Associated ROS Production under ER Stress

The mitochondrial oxidative phosphorylation and electron transport system actively participates during mitochondrial ROS generation. Studies have also indicated that ER stress events are correlated with the mitochondrial ROS production mechanisms within cells.

#### 2.4.1. ER Stress-Induced Mitochondrial ROS: Roles of GSH

The mitochondria contribute significantly to lethal levels of ROS during sustained ER stress. Preventing mitochondrial ROS accumulation completely blocks cell death, but not the ER stress response. Although ER initiates ROS generation due to ER stress, ROS subsequently affects the mitochondrial electron transfer system, amplifying mitochondrial ROS especially during severe/sustained ER stress.

Depletion of GSH (through disulfide bond reduction during ER stress) allows ROS to be generated by the mitochondria, contributing to cell death [27,28]. ROS originating from mitochondria may be derived from leaky electrons from the electron transfer system. Mitochondria-associated ROS may in turn enhance further the ER stress response, thereby amplifying mitochondrial ROS accumulation, suggesting a potential signaling mechanism of severe/sustained ER stress-associated ROS and mitochondrial dysfunction [29]. Apart from GSH, recent studies have also demonstrated that the pyroredoxin IV enzymes present in the ER lumen can abrogate or nullify H_2_PO_2_ formed during disulfide bond formation [30]. Similar to GSH, this enzyme is also involved in regulation of oxido/reduction. Therefore, maintaining the oxido/reduction mechanism may play a regulatory role in the production of ER stress-associated ROS. In summary, this information corroborates the notion that ER stress initiates oxidative stress primarily in the ER lumen consequential to protein folding or misfolding and the depletion of GSH levels (due to excessive utilization of GSH) resulting in ROS amplification in the mitochondria.

#### 2.4.2. ER Stress-Induced Mitochondrial ROS: Role of the Mitochondrial Electron Transfer Chain (ETC) System

The role of mitochondria in ROS generation during ER stress has been well reported. Interference of mitochondrial respiration significantly decreases UPR-induced ROS accumulation [31]. This phenomenon has been observed in cells treated with tunicamycin, a chemical ER stress inducer, in Perk^−/−^ cells or Nrf2^−/−^ fibroblasts, and in yeasts expressing ERV29, a stress inducing gene required for ER associated degradation [32,33]. Similarly, cytochrome *c* null cells show an attenuated response to hypoxic ROS-triggered UPR induction [34]. ER and mitochondria seem to be tuned during ER stress through the following methods or factors. Among all, the first factor is location, *i.e.*, close proximity between mitochondria and ER supporting the direct physical interaction between the two organelles [35]. The second factor is alternation in Ca^2+^ regulation [8,31] affecting mitochondrial membrane potential, ATP depletion, and ROS formation [22]. The third factor is the specific expression of inducible signals, such as Lon protease, which protects mitochondria by interfering with cytochrome *c* oxidase complex assembly/degradation, and similarly, leads to the induction of NIX (a Bcl-2 family protein that regulates ER/Sarcoplasmic Reticulum (SR) Ca^2+^ and mitochondrial membrane potential) and opening of the mitochondria permeability transition pore (MPTP) [36,37]. Therefore, a burst of oxidative stress in the ER lumen initiated by ER stress signals targets the mitochondria to further enhance production of ROS, either by increasing cytosolic Ca^2+^ or by depleting ATP, triggering oxidative phosphorylation inside the mitochondria to maximize ROS production above threshold levels.

### 2.5. Calcium-Related ROS Generation in ER Stress

Cells have evolved a sophisticated mechanism of intracellular signaling based on localized changes in the oxidation state of specific proteins. Since the internal environment of cells is a highly reducing state, an increase in the oxidative state can act as an intracellular trigger. Oxidative stress due to enhanced ROS levels causes a Ca^2+^ influx into the cytoplasm from the extracellular environment through membrane-linked channels or from the ER/SR through the ER/SR-localized channels, respectively. An increase in Ca^2+^ concentrations in the cytoplasm promotes Ca^2+^ influx into the nuclei and mitochondria [10]. Mitochondrial Ca^2+^ loading can stimulate mitochondrial metabolism and subsequently increase generation of ROS. The high mitochondrial ROS level initiates a sequence of events in which oxidative stress increases the probability of Ca^2+^ release from ER [38]. The very close proximity between ER and mitochondria leads to the accumulation of Ca^2+^ in the mitochondria [39].

#### 2.5.1. Correlation between ER Stress-Induced Ca^2+^ Release and ROS in ER

ER oxidative stress affects Ca^2+^ release from the ER. GSH and xanthine/xanthine oxidase-induced oxidative stress facilitates Ca^2+^ release from ER through inositol trisphosphate receptor (IP3R) [40]. Since thiols also regulate MPTP opening, it is likely that the redox sensitivity of IP3R and MPTP enhances oxidative damage by generating positive feedback to accelerate mitochondrial ROS production and to increase ER Ca^2+^ release and mitochondrial Ca^2+^ loading [41,42]. This process may generate more ROS and target the mitochondria for the opening of MPTP [43]. ROS has also been suggested to act on Ca^2+^ release from ER/SR by cyclic-ADP ribose (cADPR) [44]. At low concentrations of ROS, *i.e.*, nM concentrations, ROS acts as a signaling molecule that enhances Ca^2+^ release by stimulating the synthesis of cADPR in concert with calmodulin-sensitized ryanodine receptor (RyR) [45]. At higher concentrations, *i.e.*, μM concentrations, ROS inhibits the function of calmodulin, thereby inhibiting RyR [45].

In addition, at the early stage of ER stress, oxidative stress causes the release of Ca^2+^ from ER, and a large portion of the released Ca^2+^ is taken up by the mitochondria. Elevation of mitochondrial Ca^2+^ concentration ([Ca^2+^]_m_) within the matrix stimulates mitochondrial metabolism, resulting in the production of ROS. ROS can further act as a feedback signal to the ER to enhance the sensitivity of Ca^2+^ release channels. This mechanism may underlie the manner by which menadione activates repetitive Ca^2+^ spiking in pancreatic cells or during prolonged ER stress condition [46,47]. ROS-dependent sensitization of the release channels may be particularly important in conditions where cells have to generate repetitive Ca^2+^ spikes for sustained ER stress-induced opening of MPTP [43]. This positive feedback loop will decline when the Ca^2+^ within the mitochondria matrix returns to the ER. Therefore, the ER/mitochondrial Ca^2+^ cycle has important implications for the ER stress signaling system. Ca^2+^ passed from the ER to the mitochondria increases metabolism, thereby enhancing the ATP supply, and enhances mitochondrial production of ROS signals ER to increase its capacity to release Ca^2+^.

#### 2.5.2. Correlation between ER Stress-Induced Ca^2+^ and ROS at Mitochondria

As discussed above, both ER stress and oxidative stress may increase leakage of Ca^2+^ from the ER lumen through generation of ROS. In Ca^2+^ disturbance studies, ER stress is highly linked with ER stress-associated ROS [8]. ER stress-induced ROS may come from the ER-electron coupling system, *i.e.*, ERO-1α and PDI, intra ER-GSSG/GSH or NADPH-dependent P450 reductase system for which the first and latter systems extend to the mitochondria via Ca^2+^ signaling.

During ER stress, ER-induced oxidative stress targets Ca^2+^ release from ER calcium stores. Ca^2+^ can be both a physiological and a pathological effector of the mitochondria, such that increases of [Ca^2+^]_m_ in the mitochondria may alter mitochondrial functions and, eventually, ROS production [48]. The increased [Ca^2+^]_m_ can stimulate the tricarboxylic acid cycle and mitochondrial oxidative phosphorylation, enhancing ROS output by stimulating the mitochondria to work faster thus consuming more O_2_ in the process. In addition, Ca^2+^ stimulates nitric oxide synthase (NOS) to generate NO, inhibiting complex IV activity, which further enhances ROS generation at Q_o_ site in complex III [10,49,50]. This signaling axis operates within a physiological concentration of NO. Furthermore, other studies also indicate that NO, together with high [Ca^2+^]_m_, can inhibit mitochondrial complex I, resulting in the release of cytochrome *c* by inducing the opening of MPTP and blocking the respiratory chain at complex III, thereby enhancing the production of ROS [51]. On the other hand, Ca^2+^ may perturb the mitochondrial antioxidant status. Mitochondrial GSH is released very early in Ca^2+^-induced MPTP opening, suggesting that a higher amount of Ca^2+^ exposed mitochondria may generate more ROS because of diminished GSH levels.

**Figure 3:**
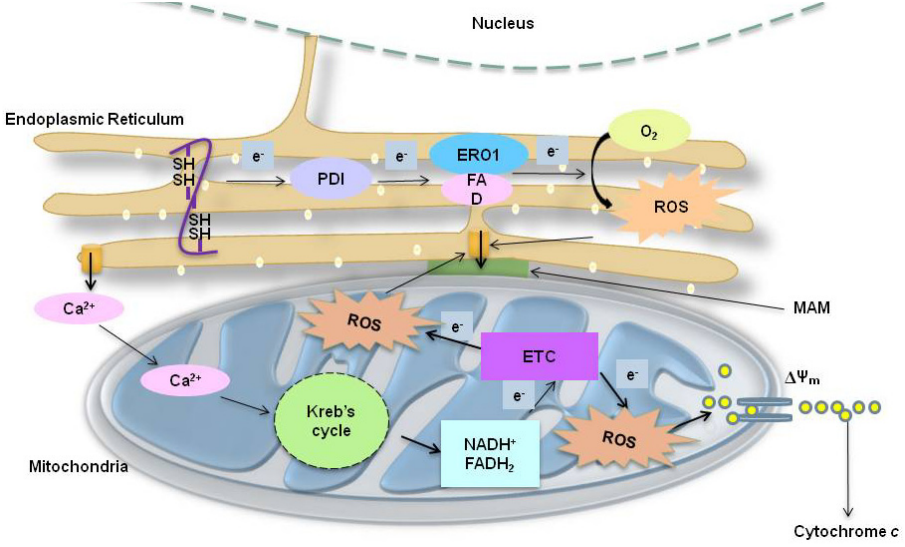
ER and mitochondrial associated reactive oxygen species (ROS) production under ER stress. ROS are generated in the ER as a part of an oxidative folding process during electron transfer between protein disulfide isomerase (PDI) and endoplasmic reticulum oxidoreductin-1 (ERO-1). ER-induced oxidative stress is further tuned for the generation of mitochondrial ROS. Ca^2+^ ions released from the ER augments the production of mitochondrial ROS which induces the Kreb’s cycle to further induce oxidative phosphorylation at the electron transport chain (ETC). Moreover, Ca^2+^ ions increase cytochrome *c* release impairing electron transfer, altering mitochondrial membrane potential and increasing the generation of ROS.

In summary, high ROS levels in the mitochondria further increase Ca^2+^ release from the ER. Furthermore, ROS can also send a feedback signal to sensitize the calcium release channel at the ER membrane (Figure 3). This may occur through ROS or reactive nitrogen species that could oxidize a critical thiol in the RyR, causing its activation and enhancing Ca^2+^ release from ER. As the antioxidative potential of the cell diminishes, the vicious cycle of Ca^2+^ release continues. ER stress can be closely associated with ER-induced oxidative stress or ER stress-triggered mitochondrial ROS, either by the induction of [Ca^2+^]_c_ or by enhancing ROS production inside the mitochondria, which further acts on Ca^2+^ release channels of the ER.

## 3. ER Stress Associated Oxidative Stress-Induced Disease

ER stress is known to be associated with a wide range of diseases including neurodegenerative disorders, stroke, bipolar disorder, cardiac disease, cancer, diabetes, and muscle degeneration. The roles of ER stress-induced oxidative stress in some of these diseases are described below. Attempts to exploit knowledge of ER stress-induced oxidative stress in various ER stress linked disease conditions are largely in their infancy, although several possible mechanisms and targets are beginning to be understood.

### 3.1. Neurodegenerative Disease

Neurons are thought to be sensitive to protein aggregates. Many reports have stated that ER stress is involved in a number of neurodegenerative diseases [52–55]. For instance, disruption of SIL1/BAP, a co-chaperone of BiP, results in accumulation of protein aggregates and neurodegeneration [56]. However, studies on ER stress-induced oxidative stress due to the UPR-regulated oxidative protein folding machinery or indirect induction of mitochondrial oxidative stress are just beginning to gain attention [33].

#### 3.1.1. Alzheimer’s Disease

Alzheimer’s disease (AD) is the most common form of dementia, resulting in progressive decline of intellectual and social abilities and productivity. AD is accompanied by a tremendous amount of cell injury and neuron loss in various parts of the brain, including the hippocampus and neurocortex [57]. AD is caused by accumulation of β-amyloid and hyperphosphorylated tau deposition in intracellular neurofibrillary tangles (NFTs) (*i.e.*, induction of ER stress) [58,59]. Autopsy studies in the brains of patients with AD suggest that the PERK-eIf2α pathway is hyperactive, [60] implying that ER stress is activated. Oxidative stress in ageing and ageing-associated disease may also underlie the pathophysiology of AD [61]. Consistent with this theory, a study has shown that AD brains exhibit ER stress-induced oxidative/nitrosative stress [62]. Thus, PDI is S-nitrosylated in the AD brain compared to control brains. S-nitrosylation of PDI facilitates further oxidation of cystein residues to sulfenic (–SOH), sulfinic (–SO_2_H), and sulfonic (–SO_3_H) acid PDI derivatives. These redox modifications enhance ER oxidative stress, inhibiting the PDI chaperone/protein folding function that leads to protein misfolding and ER stress. In AD, β-amyloid aggregation induces ER stress, altering ER and mitochondrial morphology and increasing ER oxidative stress [63]. This condition aids the upregulation of the mitochondria associated ER membrane (MAM) function at the ER-mitochondrial interface and enhances the cross-talk between the two organelles [64]. Moreover, it could also result in the dissipation of mitochondrial membrane potential, further increasing the production of ROS. Galantamine, an ER stress protective agent, has been shown to block ROS production [65]. Antioxidants such as vitamin E (α-tocopherol), vitamin C, and β-carotine, have good records of decreasing free-radical-mediated damage caused by toxic chain reaction in neuronal cells and of helping to prevent Alzheimer’s disease in mice as well as humans [66–68].

#### 3.1.2. Parkinson’s Disease

Parkinson’s disease (PD) is second only to AD in the prevalence of neurodegenerative disorders. This disease has been characterized by selective loss of dopaminergic neurons and the presence of parkin, α-synuclein, and ubiquitin accumulation in Lewy bodies [69,70]. Patients with juvenile-onset Parkinson’s disease show hereditary mutations in the ER-associated E3 ubiquitin ligase Parkin [71]. This Parkin has also been closely associated with ER-stress-induced cell death. Accumulation of misfolded proteins during PD induces ER stress thereby upregulating UPR [72]. ER stress can lead to oxidative damage by inducing the function of oxidative protein folding enzymes such as ERO1. This enzyme participates in protein disulfide bond formation during protein refolding in the ER in order to relieve ER stress. As stated above, more ROS is produced during this process. Egawa *et al.* have elucidated the relationship between ER stress and oxidative stress during PD [73]. This study has shown that ATF6a, an ER-membrane-bound transcription factor, has been shown to be activated by protein misfolding in the ER in order to protect dopaminergic neurons from MPTP. In addition, Huntington’s disease, prion-based diseases, polyglutamine disease, transmissible spongiform encephalopathies, amylotropic lateral sclerosis and neuronal storage disease are other neurodegenerative disorders in which ER stress plays a role in their pathophysiology. Further studies are required to identify the mechanism by which ER stress-induced ROS influences the development of these diseases. Antioxidants such as dibenzoylmethane (DMB) derivative compounds are identified as ER stress regulators and novel neuroprotective agents by Takano *et al*. The DMB derivative 14–26 (2-2′-dimethoxydibenzoylmethane) showed neuroprotective properties in a 6-hydroxydopamine lesion mouse model of PD [74].

#### 3.1.3. ER Stress and Age-Related Macular Degeneration

Age-related macular degeneration (AMD) is a leading cause of visual impairment in the elderly [75]. Misfolded protein induced ER stress in the retinal pigmented epithelium and/or choroid could lead to chronic oxidative stress, complementing deregulation and AMD [76]. Induction of UPR and oxidative stress enhance the production of pro-inflammatory mediators including prostaglandins, leukotrines and tumor necrosis factor α [77] and the de-repression of nuclear factor kappa B [78]. ER stress and oxidative stress can also activate systemic and local inflammation cascades directly implicated in AMD pathogenesis [79]. There is also a report of oral antioxidants used for the treatment of AMD [80]. A study was conducted in over 3000 subjects, observing the effects of high doses of antioxidants including B-carotene, vitamin C and E along with zinc. The outcome was an odds-risk reduction of 33% in the highest risk group of developing progression of severe AMD. Apart from this, addition of antioxidants such as α-tocopherol, lycopene, zeaxanthin and lutein assisted to significantly reduce the lipofuscin content in cultured retinal pigment epithelium (RPE) cells [81].

### 3.2. Cardiovascular Disease

The role of ER stress in heart diseases is quite well known. Pressure overload by transverse aortic constriction induces the expression of ER chaperones and ER stress in cardiomyocytes [82]. Since cellular Ca^2+^ homeostasis is also crucial for both ER and cardiomyocyte-specific functions, ER stress events and cardiovascular diseases are very closely related.

#### 3.2.1. ER Stress and Cardiac Hypertrophy and Heart Failure

Heart failure and cardiac hypertrophy are closely related to ER stress [83]. Morphological development of the ER is one of the characteristic histological findings during heart failure [84]. Hypoxia, oxidative stress and enhanced protein synthesis in failing hearts could all potentiate ER stress. A marked increase in GRP78 and XBP-1 expression suggested that UPR activation was associated with the pathophysiology of heart failure in humans [82,85]. Recently, it has been demonstrated that the sarco/endoplasmic reticulum calcium-ATPase isoform 3f (SERCA3f) is up-regulated in failing human hearts [86]. Interestingly, overexpression of SERCA3f induced ER stress and regulated the release of Ca^2+^ from the ER. Meanwhile, treatment with a calcium ATPase inhibitor, thapsigargin, in wild-type and transgenic mice with cardiac-specific overexpression of an active mutant of Akt (MyAkt) caused Ca^2+^ release from the heart and compromised echocardiographic parameters, (*i.e.*, elevated left ventricular end-systolic diameter and reduced fractional shortening), suppressed cardiomyocyte contractile function and intracellular Ca^2+^ handling, and enhanced carbonyl formation, superoxide production, NADPH oxidase expression and mitochondrial damage [87]. In another study, treatment with an β-adrenergic receptor (β-AR) blocker in mice with cardiac hypertrophy and heart failure reduced mortality [88]. Chronic sympathetic hyperactivity in heart failure causes sustained β-AR activation, which can deplete Ca^2+^ in the ER leading to ER stress. β-AR blockade significantly suppresses overactivation of CaMKII in failing hearts, thus inhibiting ER stress and cardiac hypertrophy [88].

#### 3.2.2. ER Stress and Atherosclerosis

Elevated triglycerides and hypercholesterolemia induces ER stress in vascular cells [89]. UPR is induced in endothelial cells by oxidized lipids, and UPR components ATF4 and XBP1 have been involved in ER-stress-induced cytokine generation through these vascular cells [90]. Similarly, addition of macrophages with cholesterol was shown to induce ER stress, enhancing expression of cytokines in presence of C/EBP homologous protein (CHOP) induction [91], which further implicates the UPR in the atherosclerosis mechanism. Abnormal deposition of free cholesterol in coronary arteries is toxic to many different vascular cell types, including macrophages, endothelial cells, and smooth muscle cells [92,93]. This condition leads to apoptosis of vascular cells, which is believed to promote atherosclerosis [94]. The generation of ROS in endothelial cells due to ER stress induction is another factor that contributes to the development of atherosclerosis. Recent studies have shown that paraoxonase 2 (PON2), an ER resident enzyme expressed in all vascular cell types, reduces ROS generation in the ER [95,96]. Similarly, treatment with a chemical chaperone phenylbutyric acid, a widely accepted ER stress protective agent, ameliorated ER stress-induced production of ROS and also ER stress during glucolipotoxicity or tunicamycin-induced ER stress in human monocytes [97].

#### 3.2.3. ER Stress and Ischemic Heart Disease

UPR markers, including expression of GRP78, XBP-1 and PDI, are induced during myocardial infarction (ischemia-reperfusion injury) in mouse hearts [98]. Conversely, induction of ER chaperones, especially GRP78, underline the phenomenon of preconditioning in the heart, in which exposure to a transient episode of brief ischemia provides subsequent protection from a sustained ischemic challenge [99]. Reduced blood flow resulting from arterial occlusion or cardiac arrest is closely associated with tissue hypoxia and hypoglycemia that cause protein misfolding and ER stress. Reperfusion of the affected tissues triggers oxidative stress, with production of NO and other reactive oxygen species that result in protein misfolding [100]. NO and other reactive molecules may also modify oxidizable residues such as cysteine in ER-associated Ca^2+^ channels including ryanodine receptors and SERCAs, causing ER Ca^2+^ depletion, yet another cause of protein misfolding [101]. Edaravone, a potent free-radical scavenger, is well-known for its protective action against lipid peroxidation. Qi *et al.* were the first to demonstrate its ability to protect cells against ER stress induced hypoxia/ischemia dysfunction by decreasing p-eIf2α and inhibiting CHOP [102].

### 3.3. Liver Disease

ER stress response also contributes to the pathogenesis of chronic viral hepatitis [103], nonalcoholic fatty liver disease [28], and alcoholic fatty liver disease [104] via hyperhomocysteinemia. Hepatic steatosis is common in patients with severe hyperhomocysteinemia due to deficiency of cystathionine beta-synthase [105,106]. Similarly, in early acute liver disease (ALD), GSH was significantly decreased due to increased GSH utilization and increased protein glutathionylation [107]. This enhances the primary pathogenic role of ER oxidative stress during the initiation of ALD via ER stress.

#### 3.3.1. Hepatitis

Hepatitis is closely related with ER stress and initiation of ER stress-induced oxidative stress. The hepatitis C virus (HCV) protein core, NS3 and NS5A have been shown to induce oxidative stress in cultured cells [108]. UPR pathways were activated including IRE1 and eIf2a phosphorylation, ATF6 cleavage and XBP-1 splicing in HCV-transgenic mice as well as HuH7.5.1 cellular system [109]. Downstream target genes including GADD34, ERdj4, p58ipk, ATF3 and ATF4 were unregulated and CHOP, a UPR regulated protein, was activated and translocated to the nucleus. HCV proteins associated with the ER membrane also induces ER stress [110]. Especially HCV replication induces transfer of high amounts of cholesterol into the ER, causing massive oxidative stress during protein folding. This ER stress-induced oxidative stress further induces Ca^2+^ release from the ER, enhancing mitochondrial Ca^2+^ uptake and the induction of mitochondrial ROS production [111]. It is known that nuclear factor-κB (NF-κB), the signal transducer and activator of transcription 2 (STAT-2) and other signaling molecules mediate these events [112,113]. On the other hand, a recent report has also demonstrated that hepatocyte Nox4 protein acts as a persistent and endogenous source of ROS in HCV-induced pathogenesis [114].

#### 3.3.2. Diabetes

Accumulating evidence suggests that ER stress plays a role in the pathogenesis of diabetes, contributing to pancreatic β-cell loss and insulin resistance [115]. β-cells have a function in secreting large amounts of insulin and other glycoproteins, so they possess an extremely well-developed ER. This secretory function of β-cells may explain why mice lacking PERK are likely to have diabetes, undergoing apoptosis of their β-cells and suffering from progressive hyperglycemia with ageing [116]. In Wolcott-Rallison Syndrome, PERK gene mutations occur where the ER cannot fold new proteins entering in it, leading to the accumulation of misfolded proteins, excessive stress generation and finally to cell death [117]. Similarly, infant-onset diabetes has been confirmed in humans as well as PERK^−/−^ mice in which patients exhibit massive β-cell loss at autopsy. Apart from this, eIf2α knock-in mice also suffer from β-cell depletion during infant stages in a more rapid manner than PERK^−/−^ mice [118]. Components of UPR in β-cells act as beneficial regulators under physiological conditions or as triggers of β-cell dysfunction and apoptosis during chronic stress. Pancreatic β-cells have an enormous capacity to synthesize and secrete insulin, rendering them vulnerable to chronic high glucose and fatty acids contributing to β-cell failure in Type 2 diabetes [119]. ER proteins, especially chaperones or folding sensor proteins that control protein folding, can also be modified by oxidation or glycation and may induce ER oxidative stress [120]. Recently, antioxidants such as MitoTempol and Mitoquinine prevented pancreatic b-cell death in cell models of glucolipotoxicity and glucotoxicity associated Type 2 diabetes [121]. Thus inhibition of oxidative stress could also prevent ER stress induced diabetes.

### 3.4. Kidney Disease

ER stress also contributes to the induction of age-related senescence phenotype, including renal fibrosis. Since ER resident proteins are highly susceptible to oxidative stress, age-related accumulation of oxidative carbonylated GRP78 or PDI causes ER dysfunction-induced diseases [122]. Similarly, the ER stress surrogate marker Grp78 was found to be highly up-regulated in immunohistochemistry profiles of kidney biopsy specimens from seven patients with UMOD-related kidney disease [123]. Oxidant-mediated protein damage is frequently observed in uremic patients. Moreover, regulation of protein damage has been suggested to predict the potential efficacy of therapeutic strategies aimed at reducing oxidative stress [124]. Similarly, Nox induces oxidative stress to assist occurrence of ER stress during renal dysfunction [125]. Apart from this, the generation of ER-induced oxidative stress is also observed during the misfolding and aggregation of coagulation factor VIII, a protein which is deficient in the ER in hemophilia A [126].

## 4. Conclusions

The mammalian cell has evolved a complex and intertwined set of signaling pathways to respond to both physiological and pathological ER stress. Although these pathways are not yet fully characterized, it is becoming clear that ER stress-induced oxidative stress and UPR are intimately involved in the pathology of various diseases. Because a number of UPR mechanisms have been implicated in many diseases, it is necessary to identify the components of ER stress that respond to targets and the particular stage of the disease by which it is activated.
